# A Case of Multiple Serositis (Pick's Disease) of Unusual Distribution

**Published:** 1921-09

**Authors:** H. J. Orr-Ewing

**Affiliations:** English Mission Hospital, Jerusalem


### A CASE OF MULTIPLE SEROSITIS (PICK'S DISEASE) OF UNUSUAL DISTRIBUTION.

BY

H. J. ORR-EWING, M.C., M.D., B.S. (Lond.), M.R.C.P.,

*English Mission Hospital, Jerusalem.*

---

CASES of multiple serositis are not common ; when met they are usually obscure in origin, and almost always give rise to considerable difficulty in diagnosis. The following case furnishes a good illustration of this obscurity and difficulty.

The patient was a Jew aged about 50, who was admitted to the English Hospital at Jerusalem in February, 1921, complaining of progressive abdominal distension. The past history (as in the majority of our patients) was probably inaccurate, but the man said he had been healthy except for malaria two years previously, when he had " much fever." He denied intemperate habits and venereal disease.

His present illness had begun two months before admission with abdominal pain and progressive distension, accompanied by dyspnœa and cough during the last month. There had been no hæmatemesis, melæna or morning vomiting, no œdema of legs, face or scrotum, and he had been free from fever lately. Six weeks previously he had been operated on in another hospital in a manner described by him thus : the stomach was opened and sewn up again, the abdomen was tapped twice and very much fluid taken away the first time, at the second time much less. The last tapping was fifteen days before admission.

The patient's appearance was that of a small man with an earthy complexion, but no jaundice. The cheeks and nose were free from rosacea. There was no pyrexia, the pulse rate was 78 to 84, the respirations 30 to 34. The abdomen was much enlarged with ascites. The scar of the operation wound was visible in the middle line of the epigastrium. There was no caput medusæ and absolutely no enlargement of the superficial veins.

The size of the liver was difficult to determine, but apparently it was very little enlarged, its dulness did not extend below the costal margin. There was tenderness over the hepatic region and a general resistance and doughy feeling beneath the abdominal wall, possibly due to rolled-up omentum. A hard, enlarged spleen extending 2½ inches below the costal margin could be felt by dipping.

The right side of the thorax appeared to be filled with fluid. The heart apex was in the fifth interspace just outside the nipple line, its action was regular and the sounds normal. The patient had a constant dry cough without expectoration. The nervous system showed no abnormalities. There was no enlargement of lymphatic glands. The urine was dark and scanty (10 to 20 ounces per diem), it contained no abnormal constituents except urates and bile pigment in excess. Specific gravity 1,028. Blood examination: Hæmoglobin, 60 per cent.; red blood corpuscles, 2,700,000; white blood corpuscles, 6,200. Differential count: Polymorphs, 65 per cent.; large lymphocytes, 8 per cent.; small lymphocytes, 25 per cent.; eosinophiles, 2 per cent. The appearance of the red cells was normal except for some crenation and poikilocytosis.

The progress of the case is seen in the following notes:—

February 20th.—Abdomen tapped. 20 oz. of straw-coloured fluid withdrawn which had a specific gravity of 1,007, contained a faint trace of albumen, but no inflammatory cells and no cancer cells. A few lymphocytes, mononuclears and endothelial cells were found.

February 22nd.—High irregular temperature commenced, which showed no periodicity. Malignant tertian rings and crescents were present in blood films. Large intramuscular doses of quinine were administered, and the temperature fell to normal by the 25th. Pyrexia, however, returned and bouts of fever continued until discharge from hospital, in spite of quinine and in spite of the fact that no more organisms were found in the blood. The attacks of fever looked almost like Pel-Ebstein paroxysms, but lacked their periodicity.

Von Pirquet reaction proved negative.

The abdomen was tapped on several occasions and refilled after each, but to a less extent each time. After tapping the liver could be mapped out, very little if at all enlarged. Until the fluid re-accumulated the dyspnœa was relieved and the quantity of urine increased.

On March 27th the man insisted on going home. There was no material alteration in his condition except that the abdomen was not so full. His treatment, apart from quinine injections and the tapping, consisted in mercurial inunction and potassium iodide internally.

The differential diagnosis in this case seems to lie between six conditions, namely tuberculosis, syphilis, cirrhosis of the liver, Banti's disease, carcinoma, Hodgkin's disease, and multiple serositis or Pick's disease.

1. *Tuberculosis* was suggested by the simultaneous infection of pleura and peritoneum. A completely negative von Pirquet reaction casts doubt on this diagnosis, although it does not entirely exclude it. I must confess that the feel of the abdomen after tapping was quite like that of tuberculous peritonitis.

2. *Syphilis.*—No Wassermann test could be made, but the pleural condition is difficult to explain as syphilitic, and there was no improvement under treatment with mercury and potassium iodide.

3. *Cirrhosis* (*multilobular*) *of the liver.*—Except for the presence of slight hæmorrhoids there was absolutely no evidence of portal obstruction. Alcohol as an ætiological factor was almost certainly absent. Moreover, if as is sometimes stated no case of alcoholic cirrhosis survives to undergo a second tapping this patient did so survive. I do not agree with this statement, as in my own limited experience I have seen a case of alcoholic cirrhosis of the liver tapped several times. But the pleural effusion is hard to explain on the cirrhosis theory.

4. *Banti's disease.*—I think the splenic enlargement was certainly malarial, but even if we concede that it may not have been due to malaria in Banti's disease, the case having reached the stage at which such marked ascites was present would inevitably have shown a much greater degree of anæmia and probably jaundice also.

5. *Malignant disease of the Peritoneum.*—This diagnosis might explain the ascites, but it would scarcely account for the left lung and pleura remaining intact whilst the right was invaded.

6. *Hodgkin's disease.*—I have seen a case of this disease in which both the pleura and the abdomen were involved, but there were enlarged glands as well to guide one to the correct diagnosis. In this case no glands were enlarged. It must, however, be recollected Hodgkin's disease without glandular enlargement is said to be a definite clinical entity.

7. *Pick's or Corcato's disease, multiple serositis.*—This is the diagnosis which I believe to be the correct one. In favour of this we have ascites, slight enlargement of the liver, perihepatitis as shown by pain and tenderness over the liver edge, the character of the exudate, a generalised involvement of the parietal and visceral peritoneum, and in addition pleural effusion. The splenic enlargement must be attributed to malaria, but the negative blood picture (except for secondary anæmia) and the normal urine are compatible with Pick's disease.

The condition is a rare one. Hale White collected 40 cases from the post-mortem records of Guy's Hospital. According to his description perisplenitis is nearly always present, and there may be ascites accompanied by chronic pleuritis, pericarditis and mediastinitis. Pleura, pericardium and peritoneum are the serous membranes most frequently involved, the rarest combination being, as in this case, pleura and peritoneum only. Hale White's account of the symptoms exactly corresponds with those in this case, "Ascites, absence of jaundice, frequent re-accumulation of fluid in the abdomen, very slight enlargement of the liver, and absence of signs of portal pressure." He makes a great point of the fact that very rarely does a case of cirrhosis live to be tapped more than once. In his opinion the usual condition is a generalised chronic peritonitis with a slight degree of associated perihepatitis not tuberculous in origin. Although in the case dealt with in this paper tuberculosis of the pleura and peritoneum cannot be definitely excluded, I think Pick's disease is the more likely diagnosis. It was probably one of the rarer forms of that rare disorder, in that the mediastinum and pericardium were quite unaffected. Treatment was of course unavailing. It always seems to me a bitter but none the less fairly true apophthegm, "Your interesting cases always die," or in more poetic rendering—

> "They answered as they took their fees,
> There is no cure for this disease."

